# Determinants and outcomes of bloodstream infections related to obesity

**DOI:** 10.1007/s10096-022-04501-9

**Published:** 2022-10-04

**Authors:** Felicity Edwards, Kate Glen, Patrick N. A. Harris, David L. Paterson, Kevin B. Laupland

**Affiliations:** 1 Queensland University of Technology (QUT), Brisbane, QLD Australia; 2 Department of Intensive Care Services, Royal Brisbane and Women’s Hospital, Level 3 Ned Hanlon Building, Butterfield Street, Brisbane, QLD 4029 Australia; 3 Department of Nutrition and Dietetics, Royal Brisbane and Women’s Hospital, Brisbane, QLD Australia; 4 Faculty of Medicine, UQ Center for Clinical Research, University of Queensland, Brisbane, QLD Australia; 5 Department of Microbiology, Pathology Queensland, Brisbane, QLD Australia; 6 Infectious Diseases Unit, Royal Brisbane and Women’s Hospital, Brisbane, QLD Australia

**Keywords:** Obesity, Bloodstream infections, BSI, Epidemiology

## Abstract

Although obesity is a major healthcare problem that is increasing in many populations worldwide, there are limited studies that have examined its contribution to infectious diseases morbidity and mortality. The aim of this study was to examine the clinical determinants and outcomes of bloodstream infections among patients with obesity. All adults within the publicly funded healthcare system in Queensland, Australia, identified with a BSI during 2017–2019 were included and the presence of obesity was based on discharge International Classification of Diseases (ICD-10) codes. Clinical features, microbiology, and outcomes were compared among obese and non-obese subjects. A total of 24,602 incident BSI were identified among 21,613 Queensland residents; of which 4,579 (21.2%) and 17,034 (78.8%) were classified as obese or non-obese, respectively. Obese patients were less likely to have community associated infections and were more likely to be younger, female, have higher comorbidity scores, and have bone and joint or soft tissue infections as compared to non-obese subjects. Obese patients had a lower proportion of *Escherichia coli* BSI and higher proportions of b-haemolytic streptococci. Although obese patients had longer hospital admissions and more repeat incident BSI within 1 year, they had lower overall case fatality. In a logistic regression model, obesity was associated with a lower risk for 30-day case fatality (adjusted odds ratio 0.51, 95% confidence interval 0.45–0.58). Obesity is associated with significant differences in the determinants and outcome of BSI. Increasing rates of obesity is likely to influence the epidemiology of BSI in populations.

## Introduction

Obesity is recognized as a major risk factor for adverse health effects and outcomes, and is increasing in many populations worldwide [1–3]. Excess body weight may predispose an individual to an increased risk of infections as a result of physiologic effects including decreased functional residual capacity, altered immunity, and chronic inflammation as well as associated comorbidities including diabetes, cardiovascular disease, chronic kidney disease, cancer, and liver disease [2, 3]. Studies conducted in critically ill populations have demonstrated that obesity is an independent risk factor for acquring central line associated infections, and that septic obese patients are more likely to have Gram-positive organisms from skin or soft tissue foci as compared to non-obese patients [4–6]. Other studies in hospitalized patients have found that obesity increased the risk for nosocomial BSI more than fivefold [7], and increased length of stay among those with candidemia [8]. It is not well defined as to whether obesity per se increases the risk of dying from serious infection [9, 10].

Although obesity is common and associated with a range of adverse health effects, there is a limited body of literature investigating obesity in relation to serious infections. Most studies have included only small numbers of subjects or have been limited to highly selected patient cohorts [7–9, 11, 12]. The objective of this study was to examine the determinants and outcomes of patients with BSI associated with obesity in a large Australian population.

## Methods

The study population comprised of all residents of Queensland, Australia, who were identified as having incident BSI and were admitted to a Queensland Health public hospital between January 1st, 2017, and December 31st, 2019. Queensland’s publicly funded health system, Queensland Health, services approximately 1.6 million episodes of care annually across 16 hospital and healthservice (HHS) regions within a population of approximately 5 million residents [13]. Approval for this study was granted with waiver of individual consent by the Human Research Ethics Committee at the Royal Brisbane and Women’s Hospital (LNR/2020/QRBW/62494).

Pathology Queensland performed all statewide blood culture testing for both community and institutional collection sites across Queensland Health. Throughout the study, all processing sites used the BACT/Alert® 3D system (bioMériux, Durham, NC) apart from the main central laboratory managing culture submissions from the Greater Brisbane Area and several rural Queensland sites which used the BACT/ALERT® VIRTUO® system (bioMériux, Durham, NC) during 2018. All blood cultures were incubated using BacT/ALERT FA plus (aerobic), and FN plus (anaerobic) media bottles for 5 days and subsequently discarded if no growth was detected.

The Queensland Health Clinical Information Systems Support Unit (CISSU) retrospectively identified all blood cultures which produced bacterial growth throughout the study period. Culture results (unique laboratory number, date and time of draw, and species identification) were obtained along with demographic (date of birth, gender, and postal code) details. Incident BSIs were differentiated by first and subsequent bacterial isolations per patient, with the first and subsequent isolation of the same species within 30 days deemed to represent the same episode of BSI. Polymicrobial infections were those where a bacterial species was co-isolated with one or more other significant pathogens within a 48-h period with two independent sets required for common contaminants in order to define significance [14].

Once incident episodes were identified, linkages with statewide databases were used to obtain additional clinical and outcome information. Encounters 2 years preceding and 1 year following an index blood culture were identified within Queensland Hospital Admitted Patient Data Collection (QHAPDC) and used to determine hospital admission and discharge dates, discharge survival status, geographical location, and ICD-10-AM diagnostic codes. The diagnosis of obesity was determined using the following codes “E660” or “E661” or “E6610” or “E6611” or “E6612” or “E6613” or “E662” or “E6620” or “E6621” or “E6622” or “E6623” or “E668” or “E669” or “E6690” or “E6691” or “E6692” or “E6693” or “U781.” For the purposes of length of stay, episodes observed within multiple continous hospital admissions (such as inter-hospital transfers) were deemed to represent a single hospital admission. The Registry of General Deaths was queried as of December 31, 2020, to confirm institution-based deaths and those occurring in other settings within Queensland. The Accessibility/Remoteness Index of Australia (ARIA +) was used to determine geographical remoteness of the study population based on their accessibilty to goods, services, opportunities, and road distance measurements from over 12,000 populated localities to the nearest Service Centres in five size categories based on population size [15].

Bloodstream infections were classified as hospital onset, healthcare associated, or community associated as previously described [16]. Comorbid illnesses were defined using the Charlson comorbidity index and were established using validated coding dictionaries [17, 18]. A clinical focus was assigned based on review of diagnosis-related group and primary hospital discharge codes.

Data was analyzed using Stata 17.0 (StataCorp, College Station, USA). Medians with interquartile ranges (IQR) were used to described non-normally distributed continuous variables and were compared using the Mann–Whitney-Wilcoxon test. Categorical data were compare using the Fisher’s exact test. A multivariable logistic regression model was developed to examine factors associated with all-cause 30-day case fatality rates; only first admissions were included in this analysis. Age, sex, onset classification, Charlson score, mono-versus polymicrobial infection, and focus of infection were included in the initial model. Stepwise backward variable elimination was performed in order to develop the most parsimonious model [19]. Calibration and discrimination were assessed using the Hosmer–Lemeshow test and the area under the receiver operator characteristic curve, respectively. In all statistical testing, *p* values of < 0.05 were deemed to represent statistical significance.

## Results

### Description of the overall cohort

There were a total of 24,602 incident episodes of BSI among 21,613 QLD adult residents; 2,239 had two, 495 had three, and 255 had four or more incident infections during the surveillance period. The overall median age was 64.3 years (IQR, 44.5–77.0), and 10,691 (43.5%) were female. The majority of BSIs were community onset (19,725; 80.1%) of which 11,224 (45.6%) were community associated, 8,501 (34.5%) were healthcare associated, and 4,877 (19.8%) were hospital onset. Overall, 5,403 (21.9%) episodes were in those classified as obese.

### Demographic determinants

**Fig. 1:**
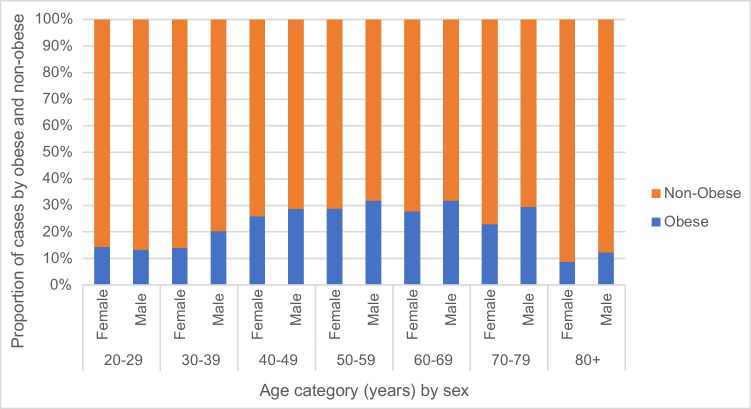
Proportion of obese vs. non-obese patients by age category and sex

There was an increasing proportion of episodes occuring among obese individuals with advancing age, peaking in the 50–59 age group and nadir in the 80 + age group. Although the distribution of obesity was similar for males and females < 30 years, more males than females were obese beyond the age of 30 years (Fig. 1). There was a significant (*p* < 0.001) difference in the proportion of obesity by remoteness although this was not across ordered groups. Geographically, the proportion of obesity was 2,786/12,832 (21.7%) for metropolitian areas, 1,570/6,115 (25.7%) for inner regional areas, 825/4,414 (18.7%) for outer regional areas, 93/529 (17.6%) for remote areas, and very remote 97/438 (22.2%).

### Clinical determinants

There were a number of different clinical features among BSIs occurring in obese and non-obese patients as detailed in Table 1. The onset location of infections was significantly different with obese patients having more healthcare related (i.e., healthcare associated and hospital onset infections; Table 1).

**Table 1:** Clinicial determinants of obese and non-obese patients with bloodstream infections in Queensland, 2017–2019

Factor	Obese (*n* = 5403)	Non-obese (*n* = 19,199)	*p*-value
Median age (IQR)	66.3 (55.2–74.5)	70.5 (55.1–81.4)	< 0.001
Male sex	2829 (52.4%)	11,082 (57.7%)	< 0.001
Median Charlson (IQR)	3 (2–5)	2 (0–4)	< 0.001
Myocardial infarction	604 (11.2%)	1560 (8.1%)	< 0.001
Congestive heart failure	1454 (26.9%)	3202 (16.7%)	< 0.001
Peripheral vascular disease	385 (7.1%)	1064 (5.5%)	< 0.001
Cerebrovascular disease	369 (6.8%)	1433 (7.5%)	0.114
Dementia	147 (2.7%)	1357 (7.1%)	< 0.001
Chronic pulmonary	931 (17.2%)	2501 (13.0%)	< 0.001
Rheumatic	97 (1.8%)	322 (1.7%)	0.553
Peptic ulcer disease	147 (2.7%)	371 (1.9%)	< 0.001
Liver disease	663 (12.3%)	2196 (11.4%)	0.092
Diabetes mellitus	2964 (54.9%)	5270 (27.5%)	< 0.001
Plegia	266 (4.9%)	936 (4.9%)	0.885
Renal disease	1687 (31.2%)	3764 (19.6%)	< 0.001
Malignancy	802 (14.8%)	4036 (21.0%)	< 0.001
HIV	3 (< 1.0%)	40 (< 1.0%)	0.018
Focus of infection			< 0.001
No focus	2755 (51.0%)	9948 (51.8%)	
Soft tissue	529 (9.8%)	1062 (5.5%)	
Bone and joint	315 (5.8%)	656 (3.4%)	
Head and neck	36 (< 1.0%)	169 (< 1.0%)	
Lower respiratory	322 (6.0%)	1453 (7.6%)	
Endovascular	166 (3.1%)	591 (3.1%)	
Central nervous system	41 (< 1.0%)	100 (< 1.0%)	
Abdominal	582 (10.8%)	2345 (12.2%)	
Urinary/pelvic	657 (12.2%)	2875 (15.0%)	
Infection onset classification			< 0.001
Hospital onset	1219 (22.6%)	3658 (19.1%)	
Healthcare associated	1962 (36.3%)	6539 (34.1%)	
Community associated	2222 (41.1%)	9002 (46.8%)	
Etiology			< 0.001
*Escherichia coli*	1240 (23.0%)	5588 (29.1%)	
*Staphylococcus aureus*	901 (16.7%)	2897 (15.1%)	
b-Hemolytic streptococci	650 (12.0%)	1212 (6.3%)	
Other Enterobacterales	388 (7.2%)	1302 (6.8%)	
*Klebsiella* species	317 (5.9%)	1280 (6.7%)	
*Pseudomonas* species	275 (5.1%)	873 (4.5%)	
Coagulase negative staphylococci	224 (4.1%)	560 (2.9%)	
Other Gram negatives	217 (4.0%)	920 (4.8%)	
Other	210 (3.9%)	874 (4.6%)	
Anaerobes	208 (3.8%)	804 (4.2%)	
*Enterococcus* species	187 (3.5%)	604 (3.1%)	
Pneumococcus	104 (1.9%)	467 (2.4%)	
Yeasts	89 (1.6%)	319 (1.7%)	
Polymicrobial	393 (7.3%)	1499 (7.8%)	

Underlying medical illnesses were common among obese patients with higher median Charlson comorbidity scores compared to non-obese. Almost one-third of non-obese patients had zero comorbidities (5,285; 27.5%), whereas obese patients with no comorbidities accounted less than one-fifth (908; 16.8%; *p* < 0.001). Charlson comorbidity scores of 1–2 were similar across both groups (1,503; 27.8% vs. 5,979; 31.1%; *p* < 0.001); however, scores of 3 + were much higher in the obese group (2,992; 55.3%) compared to the non-obese group (7,935; 41.3%; *p* < 0.001).

Overall, the most common comorbid medical conditions were diabetes, renal disease, cogestive heart failure, and malignancy; however, the comorbidities varied greatly when comparing the obese vs. non-obese cohorts. Rates of renal disease and congestive heart failure were higher in obese patients, and rates of diabetes were twice as common than the non-obese group. The rates of malignancy were higher in the non-obese group and accounted for approximately one-fifth of the cohort (Table 1).

The distribution of infectious foci was different with obese patients having a greater proportion of soft tissue infections and bone and joint infections (Table 1). The etiology also demonstrated significant variability with obese patients having relatively twice as many infections due to b-hemolytic streptococci and fewer *Escherichia coli* as shown in Table 1.

Soft tissue infections were comparably higher among obese males than obese female (301; 56.9% vs. 228; 43.1%; *p* = 0.005). Bone and joint infections were also more frequently obesity associated among males as compared to females (193; 61.3% vs. 122; 38.7%; *p* = 0.002), although this did not associate with any age group.

### Hospital admission and outcome

The median duration of hospital stay was 10 (IQR 6–25) days and the median length of hospital stay for obese patients was 3 days longer than non-obese patients (12; IQR 6–29 vs. 9; 5–21). Among the obese cohort, those with community-onset BSI, the median length of stay was 2 days longer than non-obese cohort (9; IQR 6–18 vs. 7; 4–14). Among patients incepted in 2017 and 2018, new incident BSI occurred within 1 year in 539 (10.0%) obese and 1,310 (6.8%; *p* < 0.0001) non-obese patients.

The crude 30-day case fatality following the initial episode was 7.5% (345/4,579) and was higher for non-obese patients (2,232/17,034; 13.1%; *p* < 0.001). After adjustment for confounding variables in logistic regression analysis, obesity was associated with a lower risk for 30-day case fatality (*n* = 21,613; area under receiver operator characteristics curve 0.7654; goodness of fit *p* = 1.0) as shown in Table 2.

**Table 2:** Logistic regression analysis of factors associated with 30-day case fatality among obese patients with bloodstream infections

Factor	Odds ratio	95% CI	*p*-value
Obese	0.51	0.45–0.58	< 0.001
Focus of infection			
No focus	1 (reference)	-	
Soft tissue	0.35	0.27–0.45	< 0.001
Bone and joint infection	0.37	0.26–0.52	< 0.001
Head and neck infection	0.51	0.25–1.01	0.054
Lower respiratory infection	1.43	1.24–1.65	< 0.001
Endovascular infection	0.98	0.75–1.28	0.889
Central nervous system infection	1.38	0.76–2.50	0.290
Abdominal infection	0.65	0.56–0.75	< 0.001
Urinary/pelvic infection	0.29	0.24–0.35	< 0.001
Polymicrobial infection	1.54	1.33–1.79	< 0.001
Onset classification			
Hospital onset	1	-	
Healthcare associated infection	0.70	0.63–0.79	< 0.001
Community associated infection	0.56	0.50–0.63	< 0.001
Age per year	1.03	1.03–1.03	< 0.001
Charlson comorbidity index	1.22	1.20–1.24	< 0.001

## Discussion

In this study, we report the determinants and outcomes of patients with BSI associated with obesity in an Australian population. We found older individuals, particularly males and those with comorbidites, are at a higher risk of developing BSI and the source of infection was more likely to be soft tissue or bone and joint in association with obesity. In addition, the obese cohort had longer hospital admissions and repeat incident infections particularly of b-hemolytic steptococci infections. However, the obese cohort had a lower case fatality. This study represents the largest cohort to date and adds to the relatively small published data on the epidemiology of obesity-associated BSI.

There are few previous studies that have documented excess risk for developing BSI associated with obesity. Buetti and collegues conducted a post hoc analysis of clinical trials and found that obesity was a significant risk factor for development of central line associated infections in ICUs [4]. Kaye et al. performed a retrospective case control study and using a prediction model found that obesity was an independent risk factor for nosocomial infections in those aged over 65 years [7]. In the current study and consistent with other studies, BSI risk increased with age with a male dominance [4, 7, 8]. Two previous studies from Norway showed increased risk of BSI with increasing BMI [11, 12]. Paulsen et al. stratified BSI cases by BMI and after adjusting for age and sex, were able to show a dose-dependent increase risk of BSI starting at BMI 30 kg/m^2^ [11]. Using a subcohort of European genotyping from the same dataset, Rogne et al. used Mendelian randomization design and showed that the genetically predicted BMI of 30 kg/m^2^ significantly increased the risk for BSI as composed to 25 kg/m^2^ in a population-based study [12]. Two other studies used BMI classification to assess BSI risk and showed that although obese males in their late 50 s to 70 s were more likely to have a BSI, this only applied in until BMI 39.99 kg/m^2^, compared to the BMI > 40 kg/m^2^ category where it appeared younger women in their late 40 s to late 50 s were more at risk [5, 9].

The increased risk for development of BSI among obese patient is likely related at least in part due to an increased risk for comorbidities that are known to be associated with excess BSI risk most notably diabetes, renal failure, and cardiovascular disease [20–22]. In contrast, maligancies and associated immune compromise may be risk factors associated with normal or low body weight. Arabi et al. reported on patients with septic shock from hospitals in Canada, USA, and Saudi Arabia and found that heart failure and diabetes were significant factors among the 850 obese patients whereas immunosuppresssive disorders were associated with non-obesity [5]. A smaller Greek study of 38 obese patients found diabetes and chronic pulmonary disease were more prevalent; however, solid organ and hematologic malignancy was also more common than the non-obese cohort, but this was not significant [6]. The degree to which obesity per se modifies risk for BSI beyond that explained by comorbidities requires further investigation.

Obese patients experienced a different hospital course and outcome as compared to non-obese patients. We observed an excess length of stay of 3 days associated with obesity. Barber et al. previously reported on 80 patients (28 obese and 52 non-obese) with first episodes of *Candida* species BSI and reported that the obese group had an excess median length of hospital stay of 6 days as compared to non-obese patients [8]. Our observation that obese patients were at higher risk for repeat incident BSIs episodes is novel. However, our observation that obese patients were at lower risk for death despite having more comorbidities, and longer hospital stay, has been observed elsewhere and termed the “obesity paradox” [2]. Studies in many disciplines have observed a J-shaped relationship between body weight and case fatality with overweight and moderate obesity being at a lower risk of mortality when compared to both underweight and severely obese [2]. However, given that obesity is associated with a wide range of adverse health effects, it is argued that this reflects a number of study biases rather than a protective effect associated with obesity [2, 4, 10, 12, 23].

Although our study benefits from a large cohort of patients, there are some limitations. First, we used discharge ICD-10 codes to identify obesity and did not use actual measured heights and weights in our evaluation. Studies from our region indicate that whilst ICD-10 codes identify obese patients, their sensitivity is low (i.e., 50%) such that many were likely included within the non-obese cohort [24]. This, however, would lead to a null bias, and therefore, our observations should be viewed as conservative. Second, our classification of obesity was binary such that we were not able to look at effects across a range of BMI classifications. Third, we only included admitted patients and there may be a bias toward admitting obese patients as compared to non-obese patients to hospital for management [10]. Fourth, as a result of our limited ascertainment of obesity among cases, we were unable to perform an analysis whereby we determined the risk for developing BSI among the Queensland obese and non-obese population as we have previously done in other studies [23]. Fifth, our logistic regression model did not include measures of severity of disease which would have likely further improved its discrimination. Sixth, altough not a limitation per se, it should be noted that as a result of our large sample size and power, some of our statistically signficant observations are likely not of clinical relevance (i.e., peptic ulver disease in Table 1). Finally, this dataset assumes weight is a static variable and there was no assessment of the effects associated with changes in weight during the study period.

## Conclusion

This study identifes that the clinical characteristics, course in hospital, and outcomes associated with BSI are different among obese and non-obese patients. This study adds to the growing body of literature documenting the adverse effects of obesity on infectious disease mortality. Increasing rates of obesity is likely to influence the epidemiology of BSI in populations, and further efforts to reduce the burden of obesity-associated infections are warranted.

## Data Availability

Data cannot be shared publicly due to institutional ethics, privacy, and confidentiality regulations. Data release for the purposes of research under Sect. 280 of the Public Health Act 2005 requires application to the Director General (PHA@health.qld.gov.au).
